# RNA Pol II Assembly Affects ncRNA Expression

**DOI:** 10.3390/ijms25010507

**Published:** 2023-12-29

**Authors:** Ana I. Garrido-Godino, Ishaan Gupta, Vicent Pelechano, Francisco Navarro

**Affiliations:** 1 Departamento de Biología Experimental-Genética, Universidad de Jaén, Paraje de las Lagunillas, s/n, E-23071 Jaén, Spain; aggodino@ujaen.es; 2 Genome Biology Unit, European Molecular Biology Laboratory (EMBL), Meyerhofstraße 1, 69117 Heidelberg, Germany; ishaan.iitd@gmail.com; 3 SciLifeLab, Department of Microbiology, Tumor and Cell Biology, Karolinska Institutet, 171 65 Solna, Sweden; 4 Instituto Universitario de Investigación en Olivar y Aceites de Oliva (INUO), Universidad de Jaén, Paraje de las Lagunillas, s/n, E-23071 Jaén, Spain

**Keywords:** transcription, RNA polymerases, ncRNAs, RNA polymerases assembly, Rtr1 CTD phosphatase, NNS termination, exosome, CUTs, SUTs, *Saccharomyces cerevisiae*

## Abstract

RNA pol II assembly occurs in the cytoplasm before translocation of the enzyme to the nucleus. Affecting this assembly influences mRNA transcription in the nucleus and mRNA decay in the cytoplasm. However, very little is known about the consequences on ncRNA synthesis. In this work, we show that impairment of RNA pol II assembly leads to a decrease in cryptic non-coding RNAs (preferentially CUTs and SUTs). This alteration is partially restored upon overcoming the assembly defect. Notably, this drop in ncRNAs is only partially dependent on the nuclear exosome, which suggests a major specific effect of enzyme assembly. Our data also point out a defect in transcription termination, which leads us to propose that CTD phosphatase Rtr1 could be involved in this process.

## 1. Introduction

Transcription is a well-studied step in gene expression. In eukaryotes, transcription is performed by three different RNA polymerases (five in plants) that act in coordination with complex transcriptional machinery [1,2,3]. Specifically, transcription mediated by RNA pol II has been widely studied because it is responsible for transcribing the majority of the genome, including all mRNAs and some non-coding RNAs (ncRNAs), such as: small nuclear RNAs (snRNAs); small nucleolar RNAs (snoRNAs); small Cajal body-specific RNAs (scaRNAs); microRNAs (miRNAs); long non-coding RNAs (lncRNAs); piwi-interacting RNAs (piRNAs); circular RNAs (circRNAs); and small interfering RNAs (siRNAs) [1,4,5,6]. In addition, the fragmentation of some of these non-coding RNAs produces other types of small regulatory RNAs, such as snoRNA-derived fragments (sdRNAs) [7].

Pervasive transcription, which refers to the idea that transcripts are not limited to clearly defined functional characteristics like genes [8], can be mediated by RNA pol II and has been reported in several organisms [9,10,11,12,13,14,15]. In budding yeast *Saccharomyces cerevisiae*, many non-coding transcripts that result from RNA pol II transcription have been identified using high-resolution omics techniques.

The ncRNAs that derive from the pervasive transcription of RNA pol II have been further classified according to their sensitivity to different decay pathways, transcription termination pathways or the specific conditions for their expression (reviewed in [16]). Of these ncRNAs, different types have been described: “cryptic unstable transcripts” (CUTs), degraded by the nuclear exosome; “stable unannotated transcripts” (SUTs) that exist stably in cells; and “Xrn1-sensitive unstable transcripts” (XUTs) that are degraded by cytoplasmic exonuclease Xrn1 [9,10,17,18,19]. While SUTs are detected in wild-type (WT) cells, CUTs and XUTs are detected mainly upon the inactivation of Rrp6 subunit of the nuclear exosome or Xrn1, respectively. In addition, “Nrd1-unterminated transcripts” (NUTs) have been described, which appear upon the depletion of the Nrd1 termination factor [20]. “Set2-repressed antisense transcripts” (SRATs) [21] and “meiotic unannotated transcripts” (MUTs) have also been reported [22]. Most non-coding transcripts have overlapping sequences [23,24], and many NUTs overlap CUTs [20], while XUTs significantly overlap SUTs and CUTs [18,23,24].

As the synthesis of non-functional ncRNAs could interfere with the correct production of protein-coding mRNAs through different mechanisms [25], cells exhibit strategies to control the production of pervasive transcripts. In yeast, the most prominent transcriptome surveillance mechanism coordinates both transcription termination and nuclear RNA degradation through the action of the Nrd1-Nab3-Sen1 (NNS) transcription termination complex, the nuclear exosome and the TRAMP complex [25,26]. Briefly in this mechanism, CUTs and other snRNAs and snoRNAs (sn(o)RNAs) terminate by the Nrd1-Nab3-Sen1 (NNS) pathway [25,27]). Nrd1, which recognises the RNA pol II phosphorylated at Ser5 residues of the carboxi-terminal domain (CTD) of the Rpb1 subunit, also recruits TRAMP4/5 (Trf4/5-Air2/1-Mtr4-polyadenylation), which adds a short poly(A) tail to its target RNAs [26,27]. This poly(A) tail targets the nuclear exosome to ncRNAs and promotes exosome function to completely degrade CUTs, while it trims sn(o)RNAs precursors to their stable mature forms [19,28]. Conversely, XUTs are poly-adenylated by the canonical poly(A) polymerase, Pap1 [18,24]; decapped by Dcp2; and finally degraded by Xrn1 in the cytoplasm [24], mainly by the nonsense-mediated mRNA decay (NMD) pathway [24,29]. SUTs are partially sensitive to the nuclear exosome and are degraded mainly in the cytoplasm by Xrn1 after being decapped by Dcp2 [24,25,30,31]. However, some SUTs are also degraded in the cytoplasm by the NMD pathway [25,32].

The NNS pathway requires not only Ser5P-CTD phosphorylation for Nrd1 recruitment [27] but also other posttranslational modifications of the CTD of Rpb1. Tyr1-CTD phosphorylation seems to mediate a pausing event that is critical for termination [33], probably in concert with Rtr1 Ser5P-CTD phosphatase [34,35,36], which could act also as Tyr1 phosphatase, as proposed by [35,37]. Notably, Ser7P-CTD co-localises closely with Nrd1 on CUTs, SUTs and sn(o)RNAs genes [38]. Interestingly in human cells, Ser7-CTD phosphorylation influences snRNA production in concert with the Rtr1 orthologue RPAP2 and the integrator complex [39,40,41].

Rtr1 and RPAP2 participate in RNA pol II assembly, which occurs in the cytoplasm before its nuclear import and its nuclear transport [42,43,44,45,46]. Furthermore, and specifically, Rtr1 is involved in the correct assembly of the Rpb4/7 dimer into complete RNA pol II in the cytoplasm, and its inactivation affects RNA pol II assembly and mRNA decay in *Saccharomyces cerevisiae* [42].

Assembly of eukaryotic RNA polymerases has been proposed as a sequential process involving the association of several subassembly modules in the cytoplasm prior their nuclear translocation [43,47]. The stabilization of the largest subunit of RNA pol II is facilitated by Rpb6 [48,49,50]; later, the stalk subcomplex (Rpb4/7) associates with the preassembled core enzyme [42,47]. In yeast, the assembly and transport of RNA pol II into the nucleus require the involvement of assembly and/or transport factors, many of which are conserved in human cells. In yeast, these factors encompass three GPN-loop GTPases (Npa3, Gpn2 and Gpn3), the R2TP-prefoldin-like complex, Rba50, Hsp90, Rtr1, Iwr1, Rtp1, Bud27 or Rbs1 [43]. Specifically, Rtr1 mediates the docking of Rpb4/7 to the RNA pol II core [42] and is essential for the interaction between Rpb1 and Rpb2 [46]. Furthermore, deletion of *RTR1* results in a defect in Rpb4 assembly, reduced RNA pol II transcription and increased mRNA stability [42]. Alterations in assembly factors or RNA pol II assembly lead to transcriptional abnormalities [43,51].

In this study, we investigated the effect of altering RNA pol II assembly on ncRNA transcription by using an *rpb1* mutant of *S. cerevisiae* carrying a mutation in the conserved foot domain of RNA pol II. We provide evidence that defects in RNA pol II assembly bring about a reduction in cryptic transcription, mainly of CUTs, a feature that is not influenced by the levels of its bidirectional open reading frame (ORF). We demonstrate that low levels of CUTs are not completely suppressed upon exosome inactivation, which suggests that a defect in ncRNA transcription is a consequence of the assembly defect of RNA pol II. Finally, we propose that a defect in transcription termination could account for the reduction in ncRNAs in the *rpb1* mutant.

## 2. Results

### 2.1. Altering Assembly of RNA Pol II Affects ncRNA Accumulation

In order to explore how the assembly of RNA pol II influences the transcription of ncRNAs, we used an *S. cerevisiae* strain with a mutation corresponding to the conserved foot domain of this complex called *rpb1-84*. This mutation affects the correct association of the Rpb4/7 dimer with the rest of the complex, which leads to changes in mRNA synthesis and stability [50,52].

**Figure 1:**
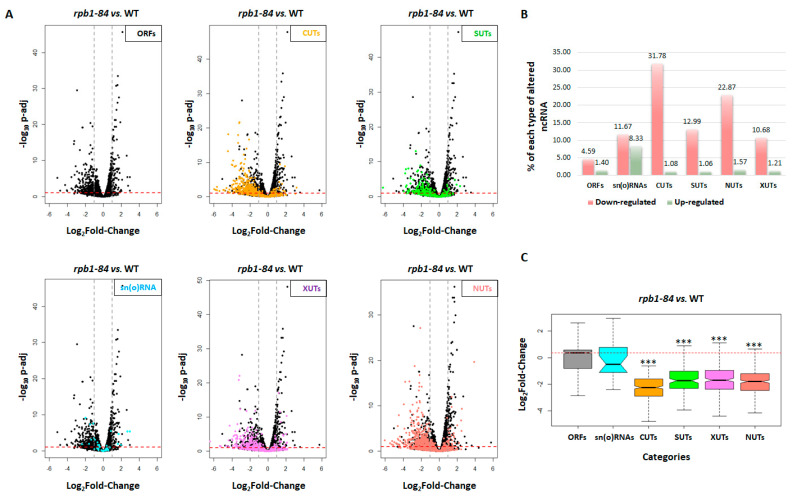
ncRNAs globally decrease by altering RNA pol II assembly. (**A**) RNA-Seq for the *rpb1-84* mutant and its isogenic wild-type (WT) strain. Volcano plots generated to identify the differentially expressed transcripts in the *rpb1-84* mutant vs. the WT strain (n = 2, DESeq2) [55]. A specific cut-off was applied to define the altered accumulation transcripts, consisting in log2 fold-change of the *rpb1-84* mutant vs. WT higher than ±1 (dashed grey lines) and *p*adj < 0.1 (dashed red line). The left upper panel shows the mRNA transcripts, whereas specific classes of ncRNAs are coloured in the other panels: CUTs (orange), SUTs (green), sn(o)RNAs (cyan), XUTs (violet) and NUTs (salmon). (**B**) Percentage of transcripts from each category that are down-regulated (red) and up-regulated (green) in the *rpb1-84* mutant vs. the WT strain according to the parameters indicated in panel (**A**). (**C**) Box-plot showing the distribution for each type of transcripts in the *rpb1-84* mutant vs. the WT strain. The indicated *p*-values were obtained from two-sided Wilcoxon rank-sum tests. *** Wilcoxon-test *p*-value < 10^−9^. Each group of analysed transcripts was compared to the ‘ORFs’ group.

To do so, a genome-wide RNA-Seq analysis was performed with two independent biological replicates in the *rpb1-84* mutant and its isogenic WT strain, grown at 30 °C in YPD-rich medium. 3′QuantSeq and an approach similar to the 3′T-Fill methodology were employed [53,54]. Each set of two replicates, which showed good similarity (see Supplemental Figure S1), were used to analyse the differentially expressed transcripts with the DESeq2 package of Bioconductor [55]. The defined annotations were used to distinguish among ORFs, CUTs, SUTs and sn(o)RNAs [10]. A specific cut-off was applied to define those genes with expression differences higher than 2-fold in the mutant compared to the WT strain (consisting of the log2 fold-change of *rpb1-84* vs. a WT higher than ±1 and *p*adj < 0.1) (Figure 1A). Regarding ORFs, 76 up-regulated and 249 down-regulated genes were identified. The functional analysis of Gene Ontology (GO) categories related to the biological processes for all differentially expressed mRNAs, using Funspec software (http://funspec.med.utoronto.ca/, accessed on 29 November 2023) [56], showed functional categories that corresponded to the stress response for the up-regulated mRNAs. The functional categories related to transcription, transcription regulation and termination were identified among the down-regulated mRNAs (Table 1). Notably, these data agreed with the previous transcriptomic analysis for *rpb1-84* and a second mutant of the foot domain of RNA pol II [52].

By applying the same cut-off for ncRNA analysis, global down-regulation was identified (Figure 1). Interestingly, while only 4.6% of ORFs were down-regulated in the *rpb1-84* mutant vs. the WT strain, 32% of the annotated CUTs and 13% of SUTs were down-regulated (Figure 1B). In addition, about 12% of sn(o)RNAs also decreased. However, a similar increase of about 8% was identified for sn(o)RNAs, while a limited increase of about 1% was observed for other ncRNAs and ORFs.

As *S. cerevisiae* ncRNAs are classified according to their sensitivity to specific decay pathways [10,17,19], we investigated the accumulation of other ncRNAs. We analysed the accumulation of XUTs, described as ncRNAs detected upon the inactivation of Xrn1 exonuclease [18,23,24] and NUTs, which are Nrd1-unterminated transcripts [20] (Figure 1). As shown, the *rpb1-84* mutant displayed a 10.68% reduction in the accumulation of the annotated XUTs, while 22.9% of the total annotated NUTs decreased in relation to the WT strain (Figure 1A,B).

Furthermore, the analyses evidenced CUTs as being the most altered class of the different types of ncRNAs in the *rpb1-84* mutant in relation to the WT strain (Figure 1C). As NUTs significantly overlapped CUTs [20], and as XUTs were 3’-extended isoforms of SUTs, and some XUTs overlapped CUTs/NUTs [23,24], we cannot rule out that part of the decrease in XUTs and NUTs could be explained by the reduction in CUTs. Accordingly, we focused on CUTs and SUTs ncRNAs.

These data suggest that affecting RNA pol II assembly decreases relative ncRNA accumulation.

### 2.2. The Decrease in CUTs Seems to Be Independent of Bidirectional Transcription

It has been described that promoters exhibit bidirectionality as an inherent property and that the frequent usage of bidirectional promoters is observed for divergent transcript pairs, which involves both ncRNAs and protein-coding genes transcription [10,20].

**Figure 2:**
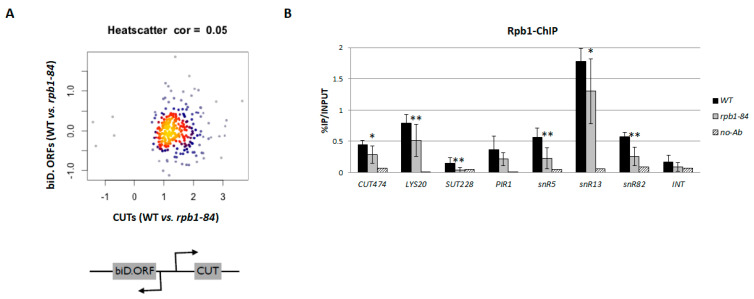
The down-regulation of ncRNAs is not dependent on bidirectional promoters. (**A**) Scatterplot showing the correlation between the accumulation of CUTs and their corresponding bidirectional sense mRNAs in the wild-type (WT) strains vs. the *rpb1-84* mutant. (**B**) Chromatin immunoprecipitation (ChIP) analysis for the different transcription units in the WT and *rpb1-84* cells, performed with an anti-Rpb1 antibody. A negative control without an antibody is also shown, as well as an intergenic non-transcribed region of chromosome V used as a negative control. The values found for the immunoprecipitated PCR products were compared as % of immunoprecipitated material (IP) vs. INPUT. Bars represent standard deviation. At least three independent experiments were performed. * *t*-test *p*-value < 0.05, ** *p*-value < 0.01.

We wondered if the promoter bidirectionality could influence the reduction in CUT accumulation. So, we analysed if the decrease in CUTs could correlate with the decrease in the expression of adjacent sense transcripts. To investigate this, we plotted the data of the gene expression between the WT and the *rpb1-84* mutant for CUTs and their corresponding adjacent ORFs (Figure 2A). The lack of correlation suggested that the drop in CUT accumulation in the *rpb1-84* mutant must not be related to an effect of bidirectional transcription.

Previous data have shown that defects in RNA pol II assembly in foot mutants lower the levels of RNA pol II occupancy in protein-coding genes [50]. Accordingly, we wondered whether the decrease in ncRNAs in the *rpb1-84* mutant could result from a differential reduction in RNA pol II occupancy in ncRNA transcription units vs. ORFs. We analysed the occupancy of RNA pol II in non-coding regions and also in their corresponding sense ORFs by chromatin immunoprecipitation using a Rpb1 antibody in the WT and *rpb1-84* mutant cells. As shown in Figure 2B, similar lower levels of RNA pol II occupancy were observed for each pair: CUT474 -*LYS20* (sense ORF) and SUT228 -*PIR1* (sense ORF) for the *rpb1-84* mutant. These data suggest that the decrease in ncRNA accumulation in relation to the mRNAs in the foot mutant did not result from the differential RNA pol II occupancy in these type of transcription units. In addition, we analysed Rpb1 occupancy in some sn(o)RNAs transcription units (snR5, snR13 and snR82), which displayed similar behaviour.

Based on the ChIP experiments, which revealed a general reduction in Rpb1 occupancy in the *rpb1-84* mutant compared to the WT strain, we speculated about the existence of additional mechanisms that govern the reduction in ncRNA accumulation in the *rpb1-84* mutant, which could be independent of RNA pol II occupancy in transcription units.

### 2.3. The Decrease in ncRNA Transcription Observed in the rpb1-84 Mutant Does Not Appear to Result from a Malfunction of Nuclear Exosome

As cryptic transcripts are eliminated from the cell by the nuclear exosome [57], we wondered whether the decrease in ncRNAs, provoked by affecting the RNA pol II assembly, could depend on nuclear exosome function.

**Figure 3:**
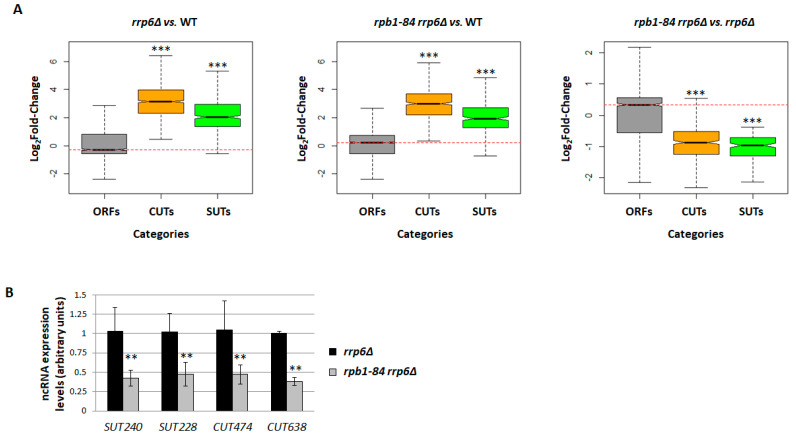
Exosome inactivation analysis by RNA-seq for the *rpb1-84 rrp6∆* double mutant, the *rrp6∆* single mutant and their isogenic wild-type strains. (**A**) Box-plots showing the distribution of ORFs, CUTs and SUTs in the *rrp6∆* mutant vs. wild type (WT, left panel); the *rpb1-84 rrp6∆* mutant vs. the WT strain (middle panel); and the *rpb1-84 rrp6∆* mutant vs. the *rrp6∆* mutant strain (right panel). The indicated *p*-values were obtained from two-sided Wilcoxon rank-sum tests. *** Wilcoxon-test *p*-value < 10^−9^. Each group of analysed transcripts was compared to the ‘ORFs’ group. (**B**) RT-qPCR analysis of CUT and SUT accumulation in the *rpb1-84 rrp6∆* vs. *rrp6∆* mutant strain. The values obtained for the *rrp6∆* single mutant are represented as 1 for all the tested transcripts. Data are shown as the mean and standard deviation (SD) of two independent experiments. rRNA 18S was used as a normaliser. ** *t*-test *p*-value < 0.01.

Therefore, we set out to analyse the effect of inactivating this pathway on the accumulation of ncRNAs in the *rpb1-84* mutant by deleting the *RRP6* gene coding for the Rrp6 subunit of the nuclear exosome [19]. For this purpose, the RNA-Seq for the *rpb1-84 rrp6∆* double mutant, the *rrp6∆* single mutant and their isogenic WT strain was also performed using the same workflow as that employed for the *rpb1-84* mutant (the similarity of replicates is shown in Supplemental Figure S1). As Figure 3A (and Supplemental Figures S2 and S3) depicts, as expected, the deletion of the *RRP6* gene led to a general increase in CUT and SUT accumulation compared to its isogenic WT strain [19]. Similar results were obtained for sn(o)RNA. In addition, the *rpb1-84 rrp6∆* double mutant also displayed a general increase in ncRNA accumulation vs. the WT strain as a consequence of the *rrp6∆* mutation with a slight drop in median levels compared to the *rrp6∆*/WT analysis (2.992 vs. 3.1411 for CUTs and 1.930 vs. 2.026 for SUTs). However, notably, a significant drop in ncRNA accumulation in the *rpb1-84 rrp6∆* double mutant strain compared to the *rrp6∆* single mutant was observed. This decrease corresponded to 11.5% of the annotated CUTs and 9% of the annotated SUTs. We also analysed the accumulation of XUTs and NUTs, and found a similar profile to that shown for CUTs and SUTs (Supplementary Figures S2 and S3).

Furthermore, the RNA-seq results were corroborated by analysing some selected transcripts by RT-qPCR (Figure 3B). Note that cryptic transcripts are often rapidly degraded, which leads to very low transcript levels under normal growth conditions [19]. Consequently, the analysis of ncRNAs in the *rpb1-84* single mutant and the WT cells led to less consistent results, which are not shown.

Taken together, these results suggest that the decrease in ncRNA accumulation in the *rpb1-84* mutant did not mainly depend on a defect in the nuclear exosome function.

### 2.4. Correcting RNA Pol II Assembly Overcomes the Decrease in Pervasive Transcription

**Figure 4:**
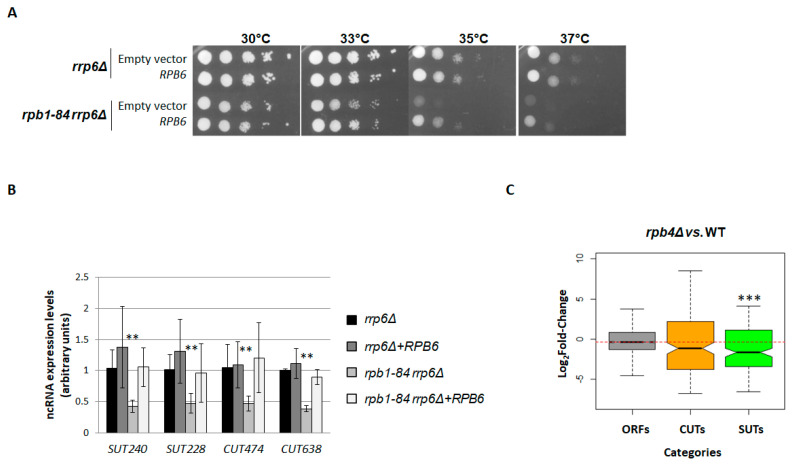
ncRNA accumulation is dependent on RNA pol II assembly. (**A**) Growth assay showing the overexpression of *RPB6* in the *rpb1-84 rrp6∆* double mutant compared to the *rrp6∆* single mutant cells grown in SD (-uracil) at different temperatures. (**B**) RT-qPCR analysis of CUT and SUT accumulation in the *rpb1-84 rrp6∆* vs. the *rrp6∆* mutant cells overexpressing *RPB6*. Data are shown as the mean and standard deviation (SD) of two independent experiments. rRNA 18S was used as a normaliser. The values obtained for the *rrp6∆* single mutant are represented as 1 for all the tested transcripts. ** *t*-test *p*-value <0.01. (**C**) Box-plot showing the distribution of ORFs, CUTs and SUTs in the *rpb4∆* mutant vs. the wild type. *** Wilcoxon-test *p*-value < 10^−9^. Each group of analysed transcripts was compared to the ‘ORFs’ group.

The defect in the correct assembly of RNA pol II in foot mutants is overcome with *RPB6* overexpression as it allows correct assembly of the dimer Rpb4/7 to the rest of the enzyme [50]. Therefore, we wondered whether the correction of the assembly of the RNA pol II by the increased dose of the Rpb6 subunit was sufficient to restore the levels of the ncRNAs in the *rpb1-84* mutant. We firstly analysed the effect of *RPB6* overexpression on the growth phenotype of the *rpb1-84 rrp6∆* double mutant and the *rrp6∆* single mutant at different temperatures. As shown in Figure 4A, the temperature sensitivity phenotype of the *rpb1-84 rrp6∆* double mutant was partially suppressed with *RPB6* overexpression, as previously described for the *rpb1-84* single mutant [50], while no additional positive effect on *rrp6∆* single mutant was observed. These data indicated that *RPB6* overexpression corrected the growth of the *rpb1-84* single mutant, but did not seem to affect the growth of the *rrp6∆* mutant.

To investigate the effect of *RPB6* overexpression on ncRNA accumulation, we performed RT-qPCR experiments in both the *rpb1-84 rrp6∆* double mutant and the *rrp6∆* single mutant containing a plasmid that allows *RPB6* overexpression or an empty plasmid as a control and analysed two SUTs and two CUTs transcription units. As shown in Figure 4B, the levels of ncRNAs were restored by *RPB6* overexpression in the *rpb1-84 rrp6∆* double mutant. These data suggested that overcoming the correct RNA pol II assembly was sufficient to suppress the pervasive transcription alteration.

The assembly defect of the *rpb1-84* mutant leads to an incorrect association of the Rpb4/7 dimer with the rest of the complex [50,52]. Therefore, to gain insights, we investigated whether lack of Rpb4 could affect ncRNA transcription. For this purpose, RNA-Seq analyses were performed for an *rpb4∆* mutant and its isogenic WT strain under the same conditions described above for the other strains used (Supplementary Figure S1 showed the similarity of replicates). The analysis of ncRNAs evidenced no major alteration to the *rpb4∆* mutant in relation to the WT isogenic strain, except for SUTs (Figure 4C and Supplementary Figure S4). These data indicate that the absence of Rpb4 does not significantly affect ncRNA accumulation.

### 2.5. The Drop in the ncRNA Levels in the rpb1-84 Mutant May Be Linked with the Alteration in ncRNA Transcription Termination

The role of Ser5P-CTD phosphatase Rtr1 in preventing the premature termination of ncRNAs by attenuating Nrd1-dependent transcription termination has been recently reported [37]. In fact, the *rtr1∆* mutant significantly down-regulates ncRNAs, which suggests increased termination at non-coding genes [37]. We have previously shown a genetic interaction between the *rtr1∆* mutant and the mutants of the foot domain of RNA pol II, including *rpb1-84* [50]. Accordingly, we hypothesise that the assembly defect of the *rpb1-84* mutant could affect ncRNA termination, leading to the increase in NNS transcription termination dependent on Rtr1. This assumption is based on the similar phenotypes observed for *rpb1-84* and *rtr1∆,* showing down-regulation of non-coding transcription [37].

**Figure 5:**
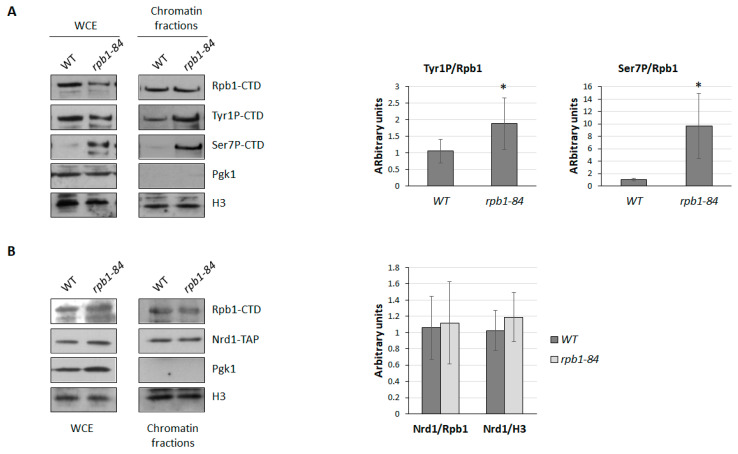
CTD-phosphorylation and the Nrd1 analysis in chromatin fractions of the foot mutant. (**A**) Left panel: whole-cell extract and chromatin-bound proteins isolated by the yChEFs procedure from the wild-type (WT) and *rpb1-84* strains grown in YPD medium at 30 °C and analysed by western blotting using specific antibodies against Rpb1, Tyr1P-CTD and Ser7P-CTD. The H3 histone was used as a positive control for chromatin and Pgk1 as a negative control for cytoplasmic contamination. Right panel: quantification of the experiments in the left panel. Levels of the phosphorylated-CTD/Rpb1 ratios for the WT strain are represented as 1. (**B**) Left panel: whole-cell extract and chromatin-bound proteins isolated by the yChEFs procedure from the WT and *rpb1-84* strains carrying an Nrd1-TAP allele grown in YPD medium at 30 °C and analysed by western blotting using specific antibodies against Rpb1 and TAP, and against the H3 histone used as a positive control for chromatin and Pgk1 employed as a negative control for cytoplasmic contamination. Right panel: quantification of the experiments in the left panel. Levels of the Nrd1/Rpb1 ratios and Nrd1/H3 ratios for the WT strain are represented as 1. At least three independent experiments with independent biological replicates were performed. * *t*-test *p*-value < 0.05.

As it has been proposed that Rtr1 could dephosphorylate Tyr1-CTD of Rpb1 [35], and as Tyr1-CTD phosphorylation has been linked with the transcription termination of ncRNAs mediating a pausing event critical for termination via the NNS pathway [33,58], we aimed to explore the phosphorylation of the Tyr1-CTD residues in the *rpb1-84* mutant in relation to its isogenic WT strain. For this purpose, we used the chromatin-enriched fractions protocol (yChEFs) and analysed chromatin-bound proteins by western blotting. As shown in Figure 5A, the Tyr1P/Rpb1 ratio in the *rpb1-84* mutant significantly increased compared to the WT strain. This increase in Tyr1P levels could increase the termination of ncRNA transcription in *rpb1-84*, consequently reducing the amount of ncRNAs.

The recruitment of the human Rtr1 orthologue, RPAP2, to snRNAs is facilitated by Ser7 phosphorylation [41], and it influences the expression of certain snRNAs [39,40,41]. It has also been proposed that Nrd1 co-localises with Ser7P in ncRNAs in *S. cerevisiae* [38]. Accordingly, we also analysed the phosphorylation of the Ser7-CTD residues in the *rpb1-84* mutant in the same samples used above. The western blot analysis of whole-cell extracts and chromatin-enriched fractions also evidenced a significant increase in Ser7P in the *rpb1-84* mutant (Figure 5A). These data collectively suggest that an altered CTD phosphorylation pattern (Tyr1P and Ser7P) could be related to the deregulation of the ncRNA expression in the foot mutant.

Furthermore, the analysis of the biological functional categories of differentially expressed genes in the *rpb1-84* mutant cells in relation to WT cells (Table 1) identified a group of mRNAs related to ncRNA transcription termination associated with the nuclear exosome. Particularly, mRNA encoding the Nrd1 subunit of the NNS complex decreased in the *rpb1-84* mutant cells (about 2-fold). Therefore, we analysed the amount of Nrd1 associated with chromatin in both the WT and *rpb1-84* mutant strains by using a Nrd1-TAP allele. However, as observed in Figure 5B, no differences in Nrd1 were found. Furthermore, Nrd1 did not seem to vary in whole-cell crude extracts, which indicates no correlation with the alteration observed during the transcriptomic analysis. In any case, we cannot rule out the possibility of partial alterations in Nrd1 occupancy in specific transcriptional units.

Collectively, these findings indicate that the assembly defect in RNA pol II provoked by the foot mutation can potentially lead to deficiencies in transcription termination during ncRNA transcription.

## 3. Discussion

This work provides evidence for the effect of altering the correct assembly of RNA pol II in the expression of ncRNAs using the *rpb1-84* mutant of Rpb1 of RNA pol II.

The *rpb1-84* mutation resides within the conserved foot domain of the major subunit of RNA pol II, Rpb1 [59], and affects the assembly of the enzyme by altering the correct association of subunit Rpb6 and dimer Rpb4/7. Altering the assembly of RNA pol II impacts transcription, which leads to a drop in mRNA levels and a general reduction in RNA pol II occupancy [50]. Similar defects in transcription have also been reported for the mutants lacking Rpb4 [52,60] or for the mutants of *RPB6*, which also affects Rpb4/7 association [61]. In agreement with the defects in mRNA expression previously reported for the foot mutant from macroarray experiments [52], our new analyses by RNA-Seq showed similar functional GO categories being altered.

Notably, our results by RNA-Seq evidenced that altering RNA pol II assembly also caused a significant down-regulation of the ncRNAs in the *rpb1-84* mutant, which was maximal for the transcripts resulting from pervasive transcription, CUTs and SUTs [10]. These data indicate that affecting RNA pol II assembly leads to many consequences in the transcription mediated by this complex. Our data also revealed a major decrease in XUTs and NUTs. However, NUTs have been suggested to be extended isoforms of CUTs and other non-coding RNAs [20], and some XUTs might overlap CUTs/NUTs [23,24]. Thus, we cannot rule out that the defect in these kinds of ncRNAs could be the result of the drop in CUT accumulation. Nor can we exclude the notion that the marked decrease in CUTs could overshadow the detection of other non-coding transcripts.

In *S. cerevisiae*, the ncRNAs that results from pervasive transcription are very low cellular abundance RNAs, particularly CUTs [10,19], and they are usually detected following the depletion of the RNA surveillance machinery that stabilises them [9,10,17,18,19]. The deletion of nuclear exosome subunits, TRAMP complex subunits and cofactors, termination NNS complex subunits and cytoplasmic exonuclease Xrn1 specifically results in an up-regulation of the different types of pervasive transcripts [10,17,18,19,23,24,31,62,63,64]. However, interestingly, the foot mutation that alters the RNA pol II assembly led to a general decrease in ncRNAs, which was clearly observed by the RNA-Seq analysis, and the inactivation of the Rrp6 subunit of the nuclear exosome in the *rpb1-84* mutant did not completely compensate for the reduction in ncRNAs. All this suggests that additional mechanisms are involved in the transcription of these types of RNAs in this mutant. It is worth noting that there are a few described conditions that bring about a drop in cryptic transcription [37,63]. Of these, one is interestingly a decrease in ncRNAs for the *rtr1∆* mutant that lacks Ser5-CTD phosphatase Rtr1 [37]. These features might be connected because a functional relation has been reported for Rtr1 and the assembly of RNA pol II [42,50] (see below).

RNA pol II assembly defects in the *rpb1-84* mutant are suppressed by *RPB6* overexpression [50]. In line with this, the increased Rpb6 dose restored the accumulation of ncRNAs, which supports the hypothesis that the reduction of ncRNAs in foot mutant *rpb1-84* is related to a defect in RNA pol II assembly. Although the defect in RNA pol II assembly in foot mutants alters the correct association of the Rpb4 subunit with the rest of the enzyme [42,50], the deletion of *RPB4* alone does not lead to a general reduction in non-coding RNA accumulation. This suggests that the decrease in ncRNAs does not seem to depend mainly on the absence of Rpb4. Accordingly, we cannot speculate that other elements are important for ncRNA transcription, which depends on correct RNA pol II assembly.

Most yeast promoters are bidirectional, which results in divergent transcript pairs that encompass both non-coding transcripts and protein-coding genes [10,20]. Furthermore, around 90% of pervasive transcripts share the 5′ NFR with a protein-coding transcript [10]. However, our data evidenced no correlation between ncRNA and ORF transcription with promoter bidirectionality. Furthermore, despite the fact that foot mutants exhibit a global reduction in RNA pol II occupancy to transcription units [50], we did not find this to be differential between the CUT transcription units and their bidirectional protein-coding gene. However, given the close proximity and potential overlap of divergent transcript pairs, the sensitivity of the chromatin immunoprecipitation technique might not be adequate for distinguishing between pervasive transcripts and their bidirectional ORFs. Based on our data and those of others who have demonstrated that either affecting the association of Rpb4 with RNA pol II or deleting *RPB4* display reduces Rpb4-mRNA imprinting, which leads to increased mRNA stability [52,65,66], we cannot rule out differential stability mechanisms operating for ncRNAs and mRNAs when RNA pol II assembly is altered.

Our data suggest a possible defect in the transcription termination of ncRNA transcription that could be related to Ser5-CTD phosphatase Rtr1, which has been shown to prevent premature termination via the NNS pathway [37]. Several pieces of evidence support this hypothesis. Firstly, Rtr1, has been described to influence Rpb4 assembly in complete RNA pol II [42], and foot mutants show an assembly defect of the Rpb4/7 dimer [50]. The location of the Rpb4/7 dimer near the CTD of the Rpb1 subunit is potentially important for its interaction with termination factors, such as Seb1 in *S. pombe* and its homologue in *S. cerevisiae*, namely the Nrd1 termination factor [67,68]. Secondly, foot mutants in combination with *RTR1* deletion are lethal [50]. Thirdly, Rtr1 has been proposed to act as the Tyr1P phosphatase of the CTD [35], and Tyr1 has been reported as being crucial for efficient ncRNAs termination via the NNS-mediated termination pathway [33]. In agreement with this, the *rpb1-84* mutant displays high Tyr1 phosphorylation levels in relation to its WT strain. Fourthly, the Rtr1 human orthologous RPAP2 has been related to the efficient transcription of non-coding RNAs, and the recruitment of RPAP2 to snRNAs is facilitated by the Ser7 phosphorylation of the CTD of Rpb1 [40]. Interestingly, foot mutants showed high Ser7 phosphorylation levels.

As previously mentioned, ncRNA transcription termination in yeast is mediated by the Nrd1-Nab3-Sen1 (NNS) pathway [27]. Interestingly, our Gene Ontology analysis of *rpb1-84* showed the down-regulation of the functional categories related to this termination pathway. Accordingly, an *rtr1∆* mutant displayed low *NRD1* mRNA levels [37]. Interestingly, as indicated above, the deletion of *RTR1* resulted in reduced ncRNA accumulation [37], similarly to that observed for the *rpb1-84* mutant. Furthermore, the human orthologue of Rtr1, RPAP2, which is knocked down by RNAi, also decreased the expression of snRNAs [40].

Nrd1 recruitment has been linked with Ser5 phosphorylation [27,33,68]. According to the role of Ser5 phosphatase [34,35,36], it has been proposed that the increased Ser5P RNA pol II CTD levels in *rtr1Δ* cells may facilitate the marked activity of the NNS-dependent termination pathway by reducing cellular ncRNA abundance [37]. In agreement with this, foot mutants also increase Ser5 phosphorylation [50]. Due to the role of Rtr1 as Tyr1P-CTD phosphatase [35] and the relation between RPAP2 and Ser7P in human cells [40], we are tempted to speculate additional mechanisms for Rtr1 to participate in NNS transcription termination, which could be related to Tyr1 and Ser7 phosphorylation. Accordingly, Nrd1 co-localises with Ser7P on ncRNA transcription units, including CUTs, SUTs and sn/snoRNAs [38], and its human orthologue, RPAP2, needs Ser7P for its recruitment to snRNA [40].

Tyr1 is necessary to display a pausing event for efficient NNS termination [33]. Furthermore, the mutations that convert Tyr1 into a non-phosphorylatable residue, such as the Y1F mutant, result in non-coding readthrough [33], which provokes a notable increase in both CUTs and SUTs. This coincides with decreased Nrd1 occupancy at the target promoters [69]. Accordingly, the increased Tyr1P of the *rpb1-84* mutant could potentially enhance NNS function and, consequently, reduce the pool of ncRNAs as a result of increased transcription termination. However, although we did not find any differences in the global amount of Nrd1, we cannot rule out that this might be observed for specific transcription units.

Our findings could potentially enhance the understanding of various human diseases. For instance, it has been described that recurrent somatic mutations in human RPB1 have been related with meningiomas [70] and pathogenic germline mutations in RPB1, some of them altering RNA pol II assembly and/or CTD length, leading to neurodevelopmental disorder [71,72]. In addition, the analysis and function of ncRNAs in human diseases represent a burgeoning field of study (reviewed in [73]). In humans, ncRNAs have been identified as significant biomarkers and mediators of cardiovascular diseases such as atherosclerosis, aneurysms and valvulopathies [74,75]. Furthermore, dysregulation of various lncRNAs plays a pivotal role in various diseases, including psoriasis [76]; different cancers like gastric, ovarian or liver cancer [77,78,79,80]; neurodegenerative diseases like amyotrophic lateral sclerosis [81] or recent COVID-19 (reviewed in [73,75]). Based on all this data, we can assert that understanding how proper RNA pol II assembly influences ncRNA transcription will enhance our understanding of certain human diseases.

In summary, we suggest that influencing RNA pol II assembly, particularly through the foot mutation, may impact ncRNA termination through the NNS pathway, likely by affecting the correct function of Rtr1, CTD phosphorylation and, possibly, by interacting with other components. Finally, further studies are necessary to uncover the mechanisms that lie behind the outcomes of RNA pol II alterations on ncRNA transcription and to identify additional elements that play a role in this process. It is important to note that altering the assembly of RNA pol II can have a broad effect on transcription, making it challenging to adequately study the specific transcription of non-coding RNAs or messenger RNAs.

## 4. Materials and Methods

### 4.1. Yeast Strains, Plasmid, Genetic Manipulations and Media

Common yeast media, growth conditions and genetic techniques were employed as previously described [82]. Yeast strains and plasmids are listed and briefly described in Supplementary Tables S1 and S2.

Strains containing the *rpb1-84* mutation along with their respective wild-type isogenic strains are derived from the wild-type strain GR21-2d (Gift from P. Thuriaux). This strain carries the deletion of *RPB1* gene, which is expressed from a 2 μm plasmid. Specific genotypes of the strains, their origin and other relevant details are listed in Supplementary Table S1.

Specifically, strain YFN542 contains the *rrp6∆::KanMX4* allele and was obtained by chromosomal integration in strain Gr21-2d of the corresponding PCR product amplified by using the genomic DNA from strain Y01777 (Euroscarf, Germany) with oligonucleotides Rrp6-501 and Rrp6-301 (Table S3) as templates. Strains YFN543 and YFN545 were derived from YFN542 through plasmid shuffle exchange employing plasmids pYEB220 and pYEB220-84, as described elsewhere [50]. Strain YFN471, containing an *rpb1-Δ187::TRP1* allele, was obtained by integrating the *TRP1* maker from plasmid pHT6 [83] into the *rpb1-Δ187::HIS3* marker of strain YFN166 through chromosomal integration.

For the dot blot growth assay, serial dilutions of yeast cultures from both wild-type and mutant strains were spotted onto minimum medium supplemented with amino acids and grown for 2–3 days at the specified temperatures.

### 4.2. RNA Extraction, Sequencing and Bioinformatic Analysis

For global RNA extraction, 50 mL cultures were grown in YPD until an OD600 of approximately 0.8 was reached. Cells were collected and utilised for total RNA extraction following a previously described procedure [84].

cDNA libraries were generated for the global expression analysis as outlined before [85]. Briefly, libraries were prepared using the QuantSeq 3′ mRNA-Seq Library Prep Kit (Lexogen, Vienna, Austria) for Illumina following the manufacturer’s instructions, and samples were sequenced with an Illumina HiSeq 2000 instrument. After obtaining the Fastq sequencing datasets with FastQC, adapters were removed using Fastx Clipper, and sequences were aligned to the *S. cerevisiae* S288C genome (SGD R64) with Hisat2. Gene counts were determined by employing featureCounts [86]. Differential RNA abundance was analysed using DESeq2 software [55].

Test statistics for box-plots were obtained from two-sided Wilcoxon rank-sum tests conducted using R software (4.2.2 version) [87].

The WT RNA-Seq used in the RNA-Seq experiment of the *rpb1-84* mutant has been previously reported [65] with GEO accession numbers GSM4603016 and GSM4603018.

The WT RNA-Seq applied in the RNA-Seq experiment of the *rpb4∆* mutant has been previously reported [85] with GEO accession numbers GSM3772984 and GSM3772985.

The other samples employed in this work are provided with GEO accession number: GSE248405.

The differential expression results of the different experiments are listed in Supplementary Table S4.

### 4.3. Reverse Transcription and qRT-PCR

First-strand cDNA was synthesised from 0.5 µg of RNA using the iScript cDNA synthesis kit (BioRad, Hercules, CA, USA) following the manufacturer’s protocol.

Real-time PCR analyses were conducted as previously outlined [85] utilising a CFX-384 Real-Time PCR instrument (BioRad, Hercules, CA, USA) and the EvaGreen detection system ‘SsoFast™ EvaGreen^®^ Supermix’ (BioRad, Hercules, CA, USA). Three independent biological replicates of each sample were analysed in triplicate to obtain a representative average. mRNA amounts were normalised to the steady-state 18S rRNA levels. The employed oligonucleotides are listed in Supplemental Table S3.

### 4.4. Chromatin Immunoprecipitation

The chromatin immunoprecipitation experiments were conducted using the Dynabeads M-280 sheep anti-rabbit (BioRad, Hercules, CA, USA) and anti Rpb1 antibody (y80; Santa Cruz Biotecnology, TX, USA) following previously described procedures [50]. Real-time PCR was carried out using a CFX-384 Real-Time PCR instrument (BioRad, Hercules, CA, USA) with SYBR premix EX Taq (Takara, Kusatsu, Japan) and the oligonucleotides listed in Supplemental Table S3. At least three independent biological replicates were analysed. For quantitative real-time PCR, a 1:100 dilution was applied for the input DNA and a 1:4 dilution for the immunoprecipitated sample DNA.

### 4.5. Chromatin-Enriched Fractions and Western Blot Analyses

The yChEFs procedure was followed to prepare chromatin-enriched fractions [88] using 50 mL of exponentially grown YPD cultures (OD600~0.6–0.8). The final chromatin-bound proteins were resuspended in 1X SDS-PAGE sample buffer, boiled and analysed by western blot with different antibodies.

Protein electrophoresis and western blots were carried out as described in [50]. The employed antibodies included the anti-Rpb1 (against the CTD; manufactured in our laboratory) [88], anti-RNA pol II CTD phosphor Tyr1 (Active Motif), anti-RNA pol II phospho-CTDSer7 (4E12) (Chromotek, Planegg, Germany), anti-phosphoglycerate kinase, Pgk1 (22C5D8; Invitrogen, Massachusetts, USA ), anti-H3 (ab1791; Abcam, Cambridge, UK) and PAP (Sigma, St. Louis, MO, USA) antibodies.

IMAGE STUDIO LITE software (5.2. version) was utilised to quantify the intensities of the immunoreactive bands.

## Figures and Tables

**Table 1:** Biological functional categories of differentially expressed genes in *rpb1-84* mutant cells with respect to wild-type cells. Analysis was performed using Funspec software [56].

	Functional Category	*p*-Value
Up-regulated	purine nucleotide biosynthetic process [GO:0006164]	7.81559 × 10^−5^
	glycolysis [GO:0006096]	0.000472358
	response to stress [GO:0006950]	0.000887445
	metabolic process [GO:0008152]	0.00134185
	gluconeogenesis [GO:0006094]	0.001615
	cell adhesion [GO:0007155]	0.00353101
	manganese ion transport [GO:0006828]	0.00466732
	‘de novo’ IMP biosynthetic process [GO:0006189]	0.00594903
Down-regulated	transcription, DNA-dependent [GO:0006351]	0.0009675
	sexual reproduction [GO:0019953]	0.00111532
	mating [GO:0007618]	0.00111532
	trehalose biosynthetic process [GO:0005992]	0.00117173
	regulation of transcription, DNA-dependent [GO:0006355]	0.0014433
	ATP-dependent chromatin remodeling [GO:0043044]	0.004667
	termination of RNA polymerase II transcription, exosome-dependent [GO:0030847]	0.00500098

## Data Availability

Data are contained within the article and supplementary materials.

## Supplementary Materials

The supporting information can be downloaded at: https://www.mdpi.com/article/10.3390/ijms25010507/s1. References [42,50,52,55,65,89,90,91,92] are cited in the supplementary materials.
