# Supplementary material for: RNA Pol II Assembly Affects ncRNA Expression
Source: Int J Mol Sci. 2023 Dec 29;25(1):507. doi: 10.3390/ijms25010507 (PMC10778713; doi:10.3390/ijms25010507)
Supplement: Supplementary file 1 [file ijms-25-00507-s001.zip › ijms-2774864-supplementary.pdf]

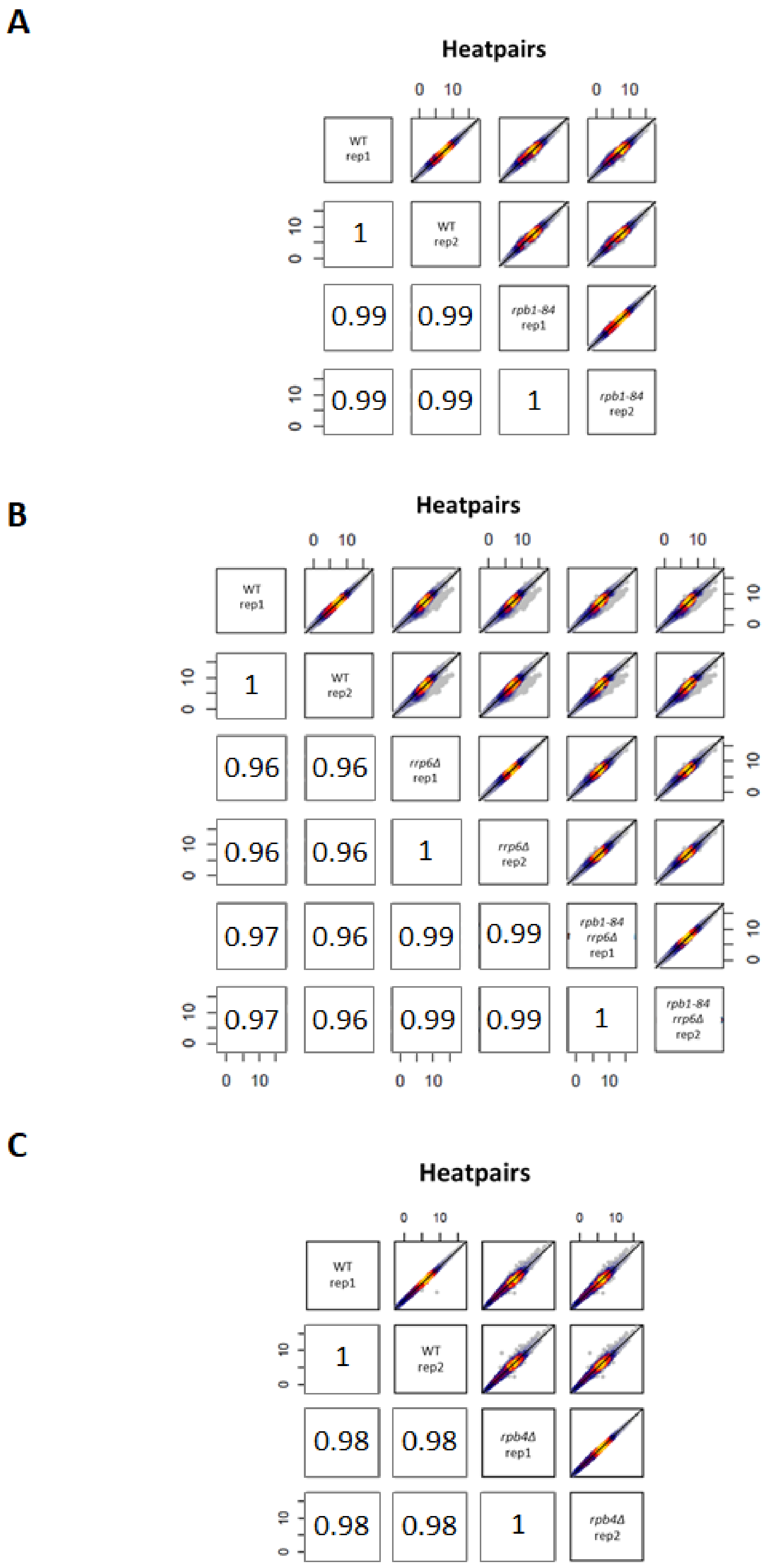


**Figure S1: Heatpairs showing a high correlation between every two biological replicates**. Similarity between samples is indicated by a numerical value, where 1 is the value of the greatest similarity, and scatterplots where the deviation of points from the trend line indicated variability between samples. Warmer colours denote the higher density of overlapping genes. Spearman rank correlation values are indicated. (A) Heatpairs of RNA-seq of the *rpb1-84* mutant and its isogenic wild-type (WT) strain. (B) Heatpairs of RNA-seq of the *rpb1-84 rrp6Δ* mutant, the *rrp6Δ* mutant and its isogenic WT strain. (C) Heatpairs of RNA-seq of the *rpb4*Δ mutant and its isogenic WT strain.

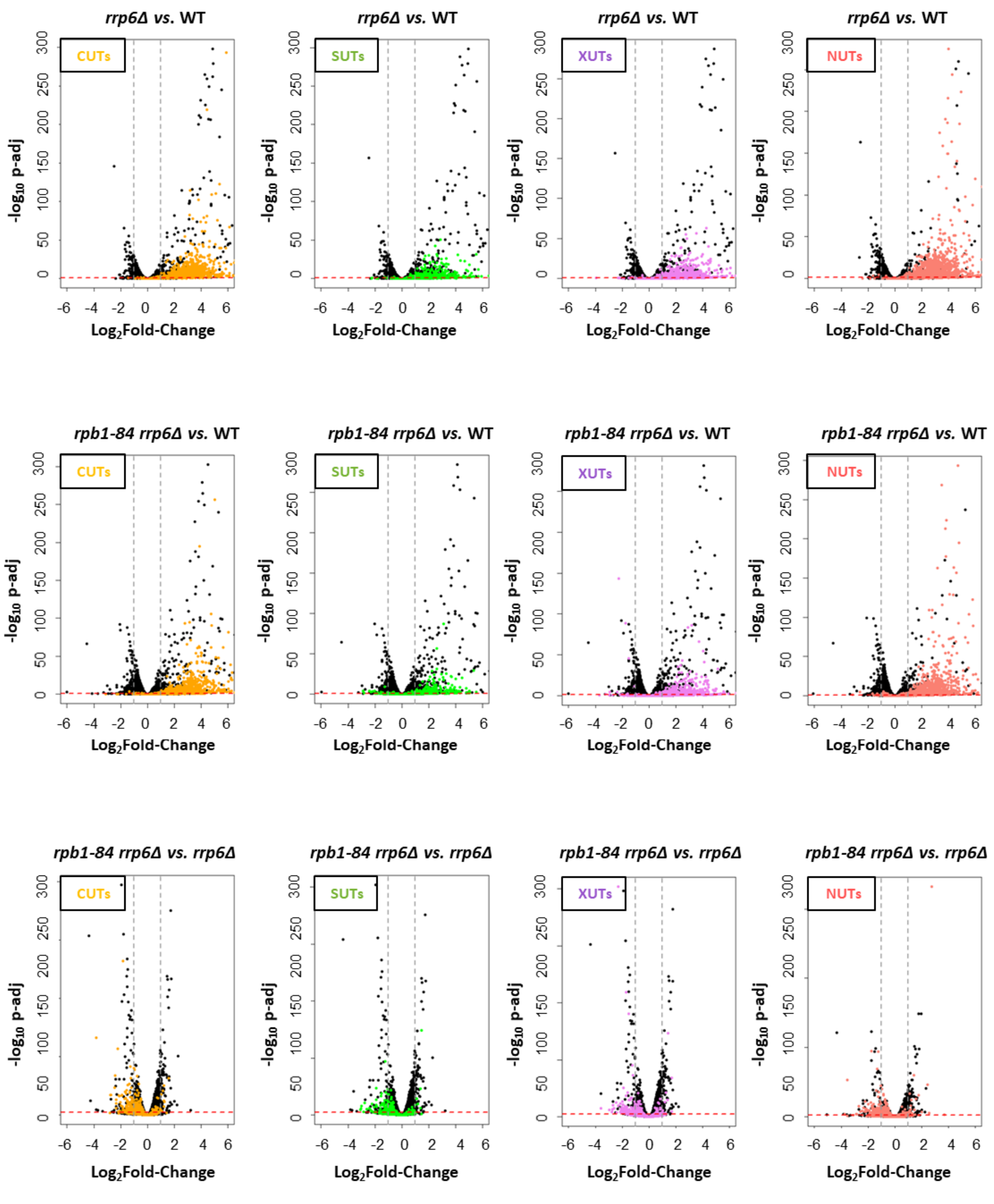


**Figure S2**

**Figure S2: ORFs and ncRNAs analysis from the RNA-seq of the wild-type strain, the *rrp6Δ* mutant and the *rpb1-84 rrp6Δ* mutant strains.** Volcano plots generated to identify the differentially expressed transcripts in the *rrp6Δ* mutant vs. the wild type (WT; upper panel), the *rpb1-84 rrp6Δ* double mutant vs. the WT strain (middle panel) and the *rpb1-84 rrp6Δ* double mutant vs. the *rrp6Δ* mutant strain (bottom panel) (n=2, DESeq2) [55]. A specific cut-off was applied to define the altered accumulation transcripts consisting in $Log_2$ Fold-Change higher than ±1 (dashed grey lines) and padj<0.1 (dashed red line). Specific classes of ncRNAs are coloured: CUTs (orange), SUTs (green), XUTs (violet) and NUTs (salmon). ORFs are coloured in black.

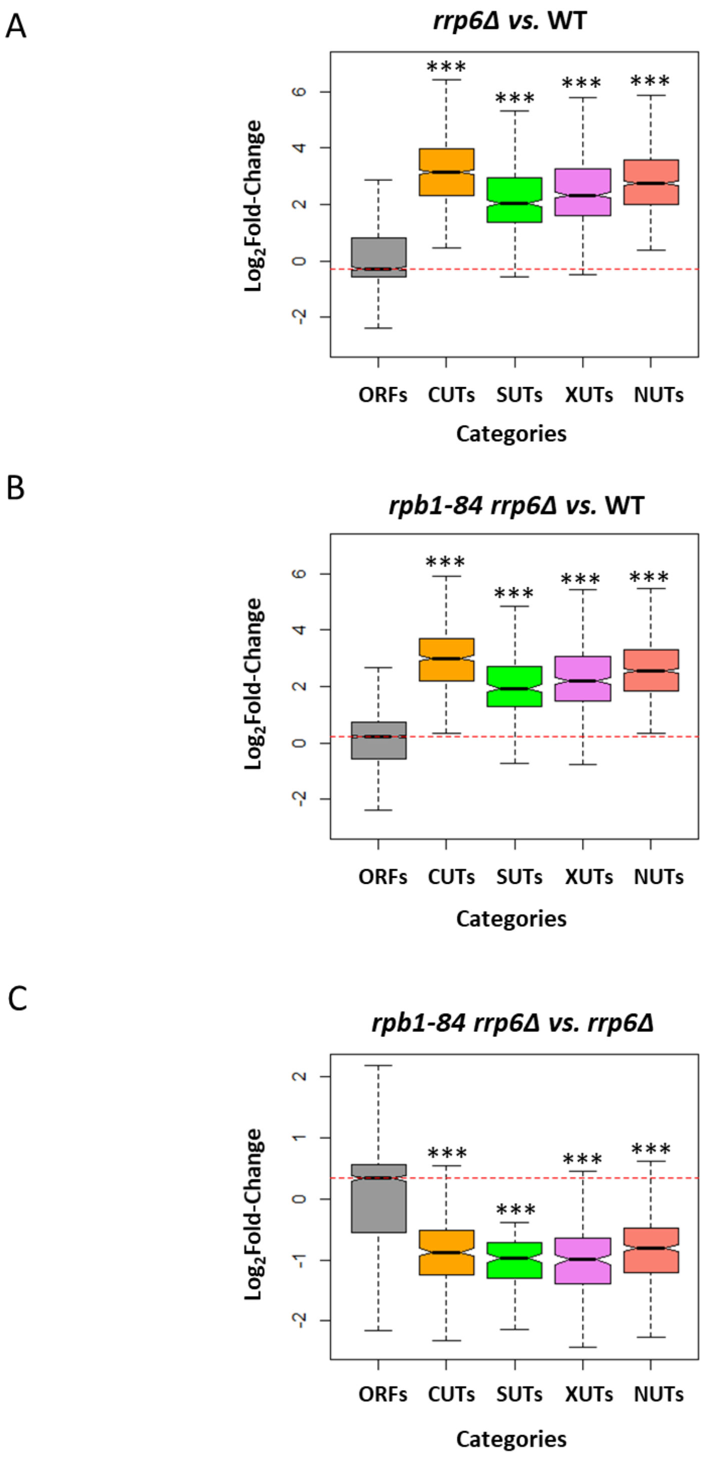


Figure S3

**Figure S3: RNA-seq of the wild-type strain, the *rrp6Δ* mutant and the *rpb1-84 rrp6Δ* mutant strains.** Box-plots showing the distribution of ORFs, CUTs, SUTs, XUTs and NUTs in the *rrp6Δ* mutant vs. the wild type (WT) (A), the *rpb1-84 rrp6Δ* mutant vs. the WT strain (B) and the *rpb1-84 rrp6Δ* mutant vs. the *rrp6Δ* mutant strain (C). The indicated p-values were obtained during two-sided Wilcoxon rank-sum test. * Wilcoxon-test p-value $< 10^{-3}$; ** p-value $< 10^{-5}$; *** p-value $< 10^{-9}$. Each group of analysed transcripts was compared to the 'ORFs' group.

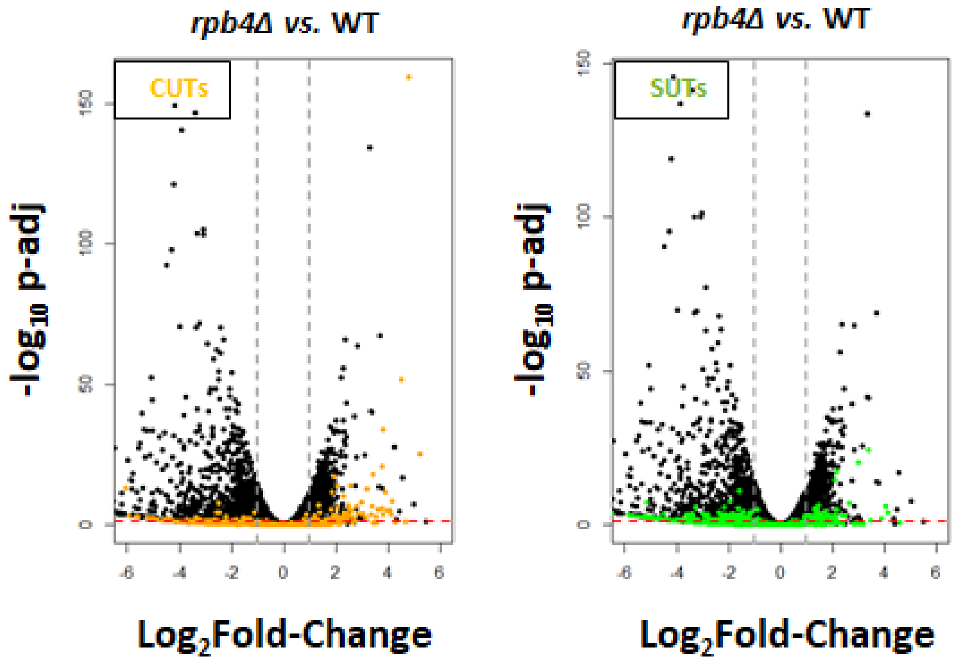


**Figure S4: CUTs and SUTs analysis from the RNA-seq of the *rpb4Δ* mutant and its isogenic wild-type strain**. Volcano plot generated to identify the differentially expressed transcripts in the *rpb4Δ* mutant vs. the wild-type (WT) strain (n=2, DESeq2) [55]. A specific cutoff was applied to define the altered accumulation transcripts consisting in $Log_2$ Fold-Change of the *rpb4Δ* mutant vs. the WT higher than ±1 (dashed grey lines) and padj<0.1 (dashed red line). Black dots show mRNA transcripts, whereas specific classes of ncRNAs are coloured: CUTs (orange) and SUTs (green).

**Table S1.** ***S. cerevisiae*** **strains**

| Strain | Genotype | Relevant information | Origin |
|---|---|---|---|
| BY4741 | *MATa his3-Δ1 leu2-Δ0 met15-Δ0 ura3-Δ0* | | Euroscarf |
| Gr21-2d | *MATα his3Δ200 leu2-3,112 trp1Δ63 ura3-52 rpb1-Δ187::HIS3 + pFL44-RPB1 (2μm URA3 RPB1)* | | P. Thuriaux |
| Nrd1-TAP | *MATa ade2 arg4 leu2-3,112 trp1-289 ura3-52 nrd1::TAP::URA3* | | [89] |
| Y01285 | *MATa his3-Δ1 leu2-Δ0 met15-Δ0 ura3-Δ0 rpb4 Δ::kanMX4* | | Euroscarf |
| Y01777 | *MATa his3-Δ1 leu2-Δ0 met15-Δ0 ura3-Δ0 rrp6Δ::KanMX4* | | Euroscarf |
| YFN116 | *MATα his3-Δ200 leu2-3,112 trp1-Δ63 ura3-52 rpb1-Δ187::HIS3 + pYEB220 (2μm LEU2 RPB1)* | | [50] |
| YFN104 | *MATα his3-Δ200 leu2-3,112 trp1-Δ63 ura3-52 rpb1-Δ187::HIS3 + pYEB220-84 (2μm LEU2 rpb1-84)* | | [50] |
| YFN161 | *MATa his3-Δ1 leu2-Δ0 lys2-Δ0 ura3-Δ0 rtr1Δ::KanMX4* | | [42] |
| YFN166 | *MATa his3-Δ1 leu2-3,112 trp1-Δ63 ura3-Δ0 rpb1- Δ187:: HIS3 + pFL44-RPB1 (2μm URA3 RPB1) rtr1Δ::KanMX4* | YFN161 X Gr21-2d | This work |
| YFN241 | *MATα his3-Δ200 leu2-3,112 trp1-Δ63 ura3-52 rpb1-Δ187:: KanMX4 + pYEB220 (2μm LEU2 RPB1)* | | [52] |
| YFN243 | *MATα his3-Δ200 leu2-3,112 trp1-Δ63 ura3-52 rpb1-Δ187:: KanMX4 + pYEB220-84 (2μm LEU2 rpb1-84)* | | [52] |
| YFN471 | *MATa his3-Δ1 leu2-3,112 trp1-Δ63 ura3-Δ0 rpb1- Δ187:: TRP1 + pFL44-RPB1 (2μm URA3 RPB1) rtr1Δ::KanMX4* | *his3::TRP1* marker disruption conversion into YFN166 strain using pHT6 plasmid [90] | This work |
| YFN518 | *MATα his3-Δ200 leu2-3,112 trp1-Δ63 ura3-52 rpb1-Δ187::HIS3 + pYEB220 (2μm LEU2* | | [65] |

| | | | |
|---|---|---|---|
| | *RPB1) RPB4::TAP::TRP1* | | |
| YFN519 | *MATα his3-Δ200 leu2-3,112 trp1-Δ63 ura3-52 rpb1-Δ187::HIS3 + pYEB220-84 (2µm LEU2 rpb1-84) RPB4::TAP::TRP1* | | [65] |
| YFN542 | *MATα his3-Δ200 leu2-3,112 trp1-Δ63 ura3-52 rpb1-Δ187::HIS3 + pFL44-RPB1 (2µm URA3 RPB1) rrp6Δ::KanMX4* | Chromosomal integration of *rrp6Δ::KanMX4* allele in strain Gr21-2d | This work |
| YFN543 | *MATα his3-Δ200 leu2-3,112 trp1-Δ63 ura3-52 rpb1-Δ187::HIS3 + pYEB220 (2µm LEU RPB1) rrp6Δ::KanMX4* | Plasmid shuffling in YFN542 | This work |
| YFN545 | *MATα his3-Δ200 leu2-3,112 trp1-Δ63 ura3-52 rpb1-Δ187::HIS3 + pYEB220-84 (2µm LEU rpb1-84) rrp6Δ::KanMX4* | Plasmid shuffling in YFN542 | This work |
| YFN668 | *MATa ade2 leu2-3,112 ura3-52 trp1-289 rpb1-D187::kanMX4 + pYEB220 (2µm LEU) nrd1::TAP::URA* | YFN241 X Nrd1-TAP | This work |
| YFN669 | *MATα ade2 leu2-3,112 ura3-52 trp1-289 rpb1-D187::kanMX4 + pYEB220-84 (2µm LEU2 RPB1) nrd1::TAP::URA* | YFN243 X Nrd1-TAP | This work |

**Table S2. Plasmids used**

| Name | | Origin |
|---|---|---|
| pYEB220 | ORI (2μm) *LEU2* | [50] |
| pYEB220-*84* | ORI (2μm) *LEU2* | [50] |
| pCM185 | ORI (CEN) *TRP1* | [91] |
| pCM185-*RPB6* | ORI (CEN) *TRP1* | [50] |
| pM647 [pRS423-SSU72] | ORI (2μm) *HIS3* | [92] |
| pHT6 HIS3→TRP1 converter | KanR; *TRP1* | [90] |

* CEN: centromeric vector; 2μm: multicopy vector

**Table S3. Oligonucleotides used**

| | |
|---|---|
| *18S*-501 | CATGGCCGTTCTTAGTTGGT |
| *18S*-301 | ATTGCCTCAAACTTCCATCG |
| *CUT474-501* | CGTGCGCTTGAGATTCAGTT |
| *CUT474-301* | AGCAAACAAGAGTCTCGGTTGA |
| *CUT638-501* | TTCAAGGACTACGAAACCACAT |
| *CUT638-301* | CCGGACGTCTATATCGCTAA |
| *CUT701-501* | CACTGAATGGGCCCTGAGG |
| *CUT701-301* | CAGGCCGAGCTTTGATTGTG |
| IntergChrV-F (intergenic region) | TGTTCCTTTAAGAGGTGATGGTGAT |
| IntergChrV-R (intergenic region) | GTGCGCAGTACTTGTGAAAACC |
| *LYS20*-501 | ATCACCGGGTTCTGTGCATT |
| *LYS20*-301 | GCGTTCCAGCCAGTTAGTCT |
| *PIR1-501* | CTGTTGCTCCAGTCTCCCAA |
| *PIR1-301* | TGCCATTAGTAATTGGAGCTGGA |
| *RRP6-501* | GCATAAGCGACAGAAAACACT |
| *RRP6-301* | AACACGCACAATATTCGAGC |
| *SUT228-501* | AACGGGATGATAGGCGCA TT |
| *SUT228-301* | CTCTTACGCTAGCTTCCCGG |
| *SUT240-501* | ACCCAAAACGCCGATCTAGG |
| *SUT240-301* | CTT TTGATAGCCAGCCCATCT |
| *snR5-501* | GCTTAAGACATCACGCCTCC |
| *snR5-301* | ACACCTAGAGCGAACCAATGA |
| *snR13-501* | GGCATCTCAAATCGTCTCTATATC |
| *snR13-301* | GAGTTTTTCCACACCGTTACTGAT |
| *snR82-501* | TGGCTCTTCAACACATTTCAACA |
| *snR82-301* | AAAACTCCCGGTAACCAGGC |

The primers used were as follows (all 5′-3′)
